# Natural and Sustainable Polymers for Regenerative Wound Care: A Review of Chitosan, Gelatin, and Cellulose

**DOI:** 10.3390/molecules31203657

**Published:** 2026-10-13

**Authors:** Hoda Motaghed, Niya S. King, Demetrius A. Finley, Roderquita K. Moore, Michael L. Curry

**Affiliations:** 1 Department of Nanoengineering, The Joint School of Nanoscience and Nanoengineering, North Carolina Agricultural and Technical State University, Greensboro, NC 27401, USA; hmotaghed@aggies.ncat.edu (H.M.); dafinley@ncat.edu (D.A.F.); 2 IMPACT Lab, Research Facility I, The Joint School of Nanoscience and Nanoengineering, North Carolina Agricultural and Technical State University, Greensboro, NC 27411, USA; 3 College of Engineering, North Carolina Agricultural and Technical State University, Greensboro, NC 27411, USA; nsking@ncat.edu; 4 USDA Forest Service, Forest Products Laboratory, Madison, WI 53726, USA; roderquita.k.moore@usda.gov

**Keywords:** wound healing, tissue engineering, chitosan, gelatin, cellulose, hydrogel, scaffold fabrication, skin regeneration

## Abstract

The field of skin tissue engineering has rapidly advanced in recent years. This advancement has been driven by the increasing need to address biocompatibility, fabrication techniques, antimicrobial properties, hemostatic properties, and porosity of the materials used for tissue repair and regeneration. This review focuses on three common materials for scaffold fabrication in skin tissue engineering, cellulose, chitosan, and gelatin. As the most common natural organic polymer on Earth, cellulose is a readily available and renewable material that is distinguished by a remarkable combination of advantages. These advantages include biocompatibility, biodegradability, renewability, and good mechanical strength, rendering it a nontoxic and environmentally friendly substance. The benefits of chitosan include its antimicrobial and hemostatic properties, while gelatin enhances cell adhesion and proliferation, contributing to the scaffold’s overall biocompatibility. The inherent porous structure of these materials supports fluid flow, cell infiltration, and nutrient transport, creating a conducive environment for tissue repair and regeneration. In addition, this review highlights improved techniques in fabricating a durable scaffold. It also addresses the challenges in fabrication, such as controlling the pore size and uniformity of the pores and structure within the scaffold. This is crucial for cell growth and tissue regeneration. These insights contribute to a deeper understanding of tissue repair and underscore the importance of using scaffolds and multilayered materials in skin tissue regeneration. Further research in skin tissue engineering will be essential for overcoming existing challenges and realizing the full potential of cellulose, chitosan, and gelatin in tissue engineering.

## 1. Introduction

**Figure 1:**
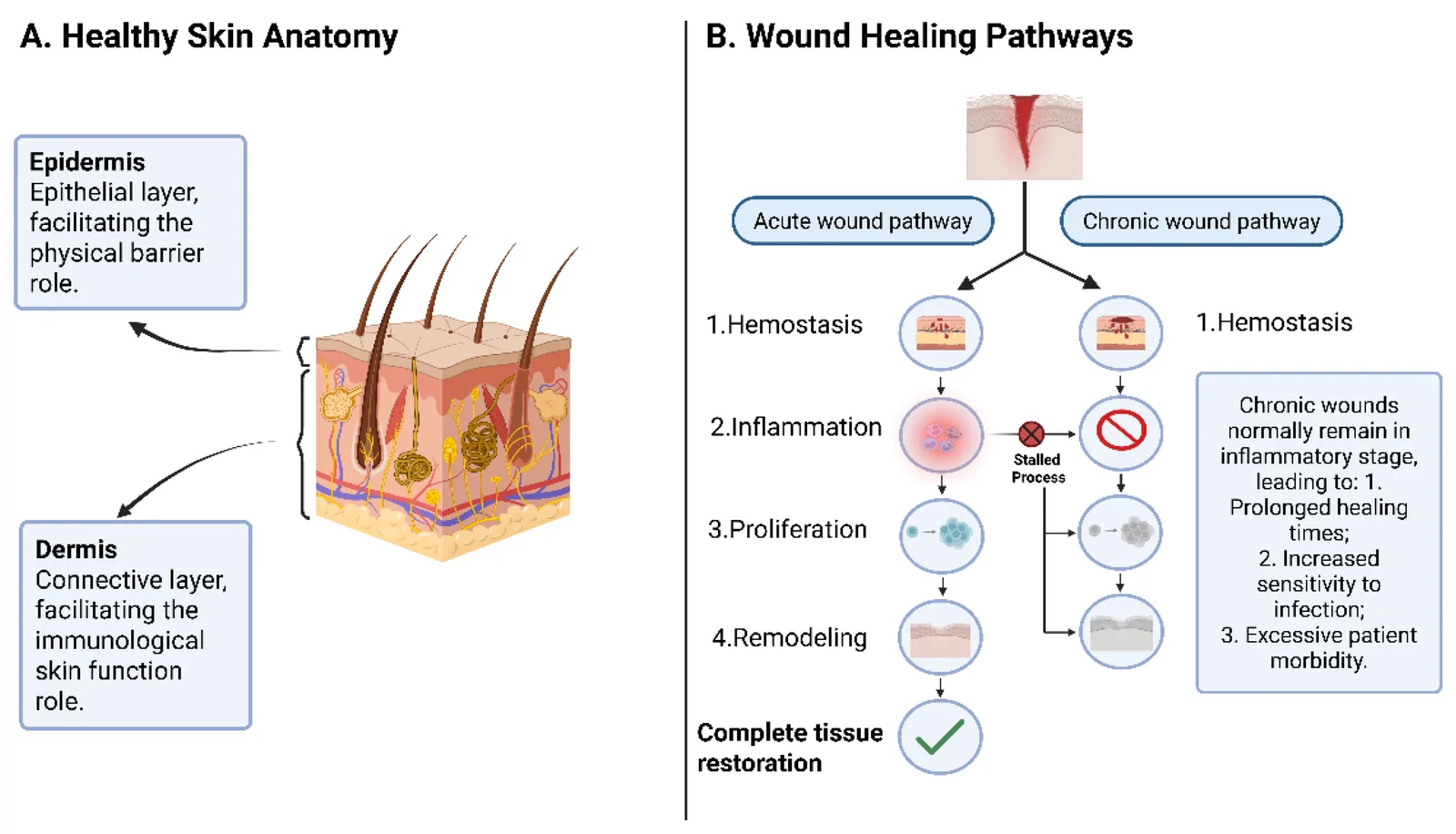
Schematic illustration of human skin anatomy and the divergent biological pathways of acute versus chronic wound healing. Created in BioRender. King, N. (2026) https://BioRender.com/2noy7qp.

Tissue engineering is a multidisciplinary field that emerges from the convergence of various scientific and technological domains, including biology, chemistry, engineering, medicine, pharmacy, and materials science. This integration of knowledge and techniques from various areas enables the development of innovative strategies to regenerate, repair, or replace damaged tissues and organs [1]. The primary goal of tissue engineering is to create functional replacements for damaged tissues or organs. Scaffolds are essential as they offer an alternative to traditional organ and tissue transplants. Their primary function is to provide a supportive structure that promotes tissue growth and cell proliferation. Biomaterials are fundamental to tissue engineering, with a wide range of natural and synthetic materials that have been investigated for scaffold fabrication [2]. Tissue engineering applications can be broadly categorized according to the target tissue for repair or regeneration, including skin [3,4], bone [5], cardiac [6], muscle [7], neural [8], and vascular tissue [9]. The skin is made up of the epidermis (i.e., the epithelial layer, which facilitates the physical barrier role) and the dermis (i.e., the connective layer, which facilitates the immunological role of the skin). Tissue-engineered skin substitutes for wound healing have evolved considerably in the last few years. New advances have been made in the development of skin substitutes made of artificial and natural materials. The most widely used natural polymers in biomedical applications are polysaccharides such as alginate, chitosan, starch, and cellulose. There are also common proteins used in biomedical applications, such as collagen, gelatin, and silk fibroin [2]. Skin wound healing, a complex biological process that involves a cascade of cellular and molecular reactions, remains an ongoing clinical problem [10]. Clinically, wounds are commonly categorized as acute or chronic based on whether they follow the expected path of repair with timely progression through the normal healing phases. Although acute wounds usually progress through well-established stages of hemostasis, inflammation, proliferation, and remodeling to achieve complete tissue restoration, chronic wounds generally remain in the inflammatory stage, leading to prolonged healing times, increased sensitivity to infection, and excessive patient morbidity [10,11]. Chronic wounds, such as diabetic foot ulcers, pressure ulcers, and venous leg ulcers, bear a large economic burden on healthcare systems around the world that require new and effective therapeutic regimens (Figure 1). The limitations of conventional wound dressings that have functioned mainly as passive barriers have emphasized the demand for advanced biomaterials capable of actively stimulating regeneration and promoting the cascade of healing [12].

Chitosan, gelatin, and cellulose have each been the subject of extensive individual reviews as wound-care biomaterials, and numerous prior reviews have addressed one or two of these polymers, typically in the context of a single fabrication method. The present review is distinguished by its integrated, comparative treatment of all three polymers across the full range of fabrication platforms used in wound-care scaffold development, namely hydrogels, electrospinning, freeze-drying, salt leaching, and 3D printing, and by its explicit discussion of the manufacturing, sterilization, reproducibility, and regulatory barriers that determine whether laboratory-scale advances can reach clinical practice. In synthesizing this literature, this review aims to move beyond a descriptive survey toward a critical evaluation that identifies where the evidence base is strong, where comparative data across studies are lacking, and what steps are needed to advance these materials toward clinical translation.

## 2. The Biology of Wound Healing and Material Requirements

Effective wound care requires a deep understanding of the intricate biological processes that govern tissue repair. Wound healing is a highly orchestrated physiological process, traditionally described as having four overlapping yet distinct phases: hemostasis, inflammation, proliferation, and remodeling [13]. It is initiated immediately upon injury by vasoconstriction and platelet accumulation, forming a fibrin clot to prevent blood loss and providing a temporary matrix for subsequent cellular invasion. This is immediately succeeded by the inflammatory stage with recruitment of neutrophils and macrophages to kill debris and pathogens. During inflammation, cytokines and growth factors are released to coordinate the subsequent stages of repair [14]. The proliferative phase is characterized by angiogenesis, granulation tissue formation, re-epithelialization, and wound contraction as fibroblasts synthesize new extracellular matrix and keratinocytes migrate to restore the epidermal barrier. Finally, the remodeling phase, lasting months to years, involves collagen reorganization, resulting in increased tensile strength and scar maturation [15,16,17].

**Figure 2:**
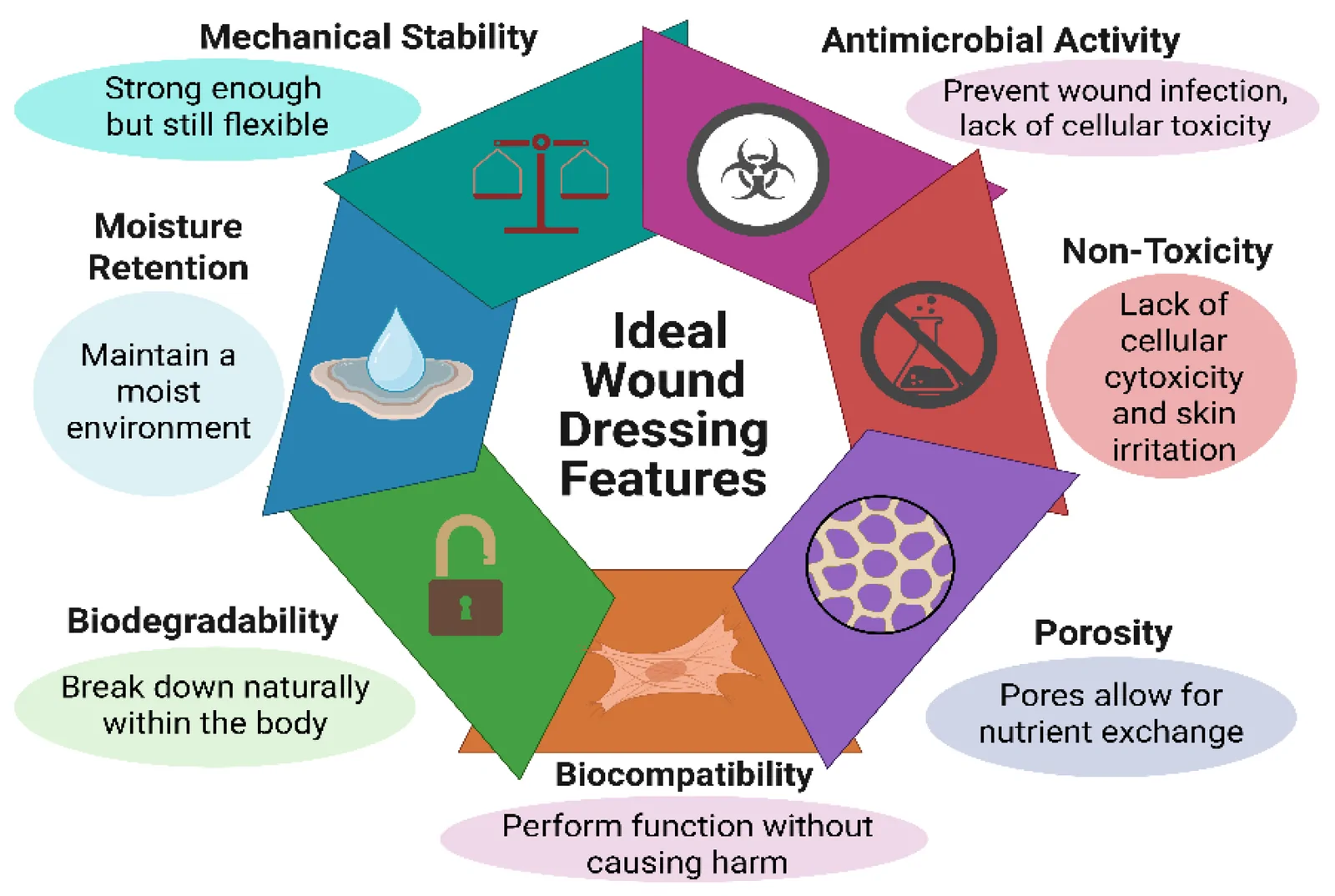
The features of the ideal wound dressing. Created in BioRender. King, N. (2026) https://BioRender.com/0mr52tk.

Given the complexity of the wound-healing cascade of biological responses, an ideal dressing should provide multiple complementary functions to support and accelerate repair. The key material requirements of the properties most significant are illustrated in Figure 2.

- **Biocompatibility:** The dressing material should be non-toxic and cause minimal adverse immune response. It should support cell viability, proliferation, and differentiation [18].
- **Antimicrobial activity:** Wounds are susceptible to microbial colonization, which can lead to infection and delayed healing. Therefore, dressings with inherent antimicrobial properties or those capable of delivering antimicrobial agents are of critical importance.
- **Hemostatic capability:** In particular for acute wounds, the dressing should help control bleeding and support clot formation to initiate the healing cascade [19,20].
- **Mechanical strength and elasticity:** The dressing should protect the wound from external forces while maintaining flexibility. Ideally, its tensile properties should be compatible with the native skin to reduce discomfort and prevent tearing, especially in dynamic anatomical sites [21].
- **Porosity and degradation rate:** An appropriate porous architecture enables gas exchange, diffusion of nutrients, and cell infiltration. In scaffold-based dressings, the size and distribution of the pores influence cell adhesion and proliferation [22]. The degradation rate should be controllable and aligned with the formation of new tissue, minimizing the need for frequent dressing changes [23].
- **Moisture retention:** Maintaining a moist environment is essential for successful wound healing, promotes cell migration, and autolytic debridement, while adequate absorption of exudate helps prevent maceration and desiccation [24].

This review focuses on three fundamental biomaterials in scaffold development: cellulose, chitosan, and gelatin. These materials are selected for their porosity, biodegradability, and synergistic potential in composite scaffolds, as well as their cost-effective production.

## 3. Biomaterials in Scaffold Development

### 3.1. Gelatin: Enhancing Cellular Interactions

The primary objective of tissue engineering is to develop scaffolding systems that provide an optimal environment for cell growth and, ultimately, support tissue repair and regeneration [25]. Among natural biomaterials, gelatin is a collagen-derived biopolymer produced by partial hydrolysis of collagen, a fibrous protein abundant in animal skin, bones, and connective tissues. Industrially, gelatin is most commonly derived from mammalian tissues (e.g., porcine skin, bovine hides, and bones), although fish- and poultry-derived gelatin are also used [26].

Gelatin can reversibly transition between the sol and gel states, which has supported its widespread use in biomedical applications, including as a hemostasis agent. Upon contact with blood, gelatin can swell and provide a matrix that promotes platelet activation and aggregation, accelerating coagulation [27]. In addition, gelatin is enzymatically degradable and generally well tolerated, with minimal immunogenicity, which supports its use in medical products [28]. In wound healing, its biocompatibility and non-toxicity are particularly valuable, as gelatin can support cell adhesion and proliferation to promote repair [29].

A key advantage of gelatin is that it can readily crosslink to form hydrogels and composite networks. This reactivity is mainly attributed to its amino (–${\mathrm{NH}}_{2}$), carboxyl (–COOH), and hydroxyl (–OH) functional groups, which participate in reactions with a range of crosslinking agents. Crosslinking can improve mechanical strength, thermal stability, and water-handling properties, making gelatin-based structures attractive for applications such as skin tissue engineering [30]. These gelatin–cell interactions help to create a microenvironment that supports key cellular activities during regeneration [31]. From a sustainability perspective, gelatin is often obtained as a byproduct of the meat industry; converting cartilage, bones, and skins into gelatin can add value to low-cost waste streams and support the development of bio-based products [32].

Beyond these foundational properties, several advances have further expanded gelatin’s role in wound-care scaffold design. Chemical functionalization of gelatin with methacrylate groups yields gelatin methacryloyl (GelMA), a photo-crosslinkable derivative that retains gelatin’s native cell-adhesion and protease-sensitive motifs while allowing precise, light-triggered control over hydrogel stiffness and degradation, making it one of the most extensively studied gelatin derivatives for tissue-engineered constructs [33]. Clinically, gelatin-based biomaterials have been applied directly to chronic, hard-to-heal wounds, where they support hemostasis, provide peri-wound antibacterial and anti-inflammatory effects, and promote vascular and epithelial regeneration [34]. In addition to hydrogels and sponges, gelatin is frequently processed into electrospun nanofiber mats that replicate the fibrous architecture of the native extracellular matrix; such gelatin-based nanofiber dressings combine high porosity and exudate-absorbing capacity with the ability to carry antimicrobial, anti-inflammatory, or pro-angiogenic agents for burn and diabetic wound applications [35].

### 3.2. Chitosan: A Biopolymer with Antimicrobial and Hemostatic Properties

Where gelatin’s contribution to wound repair centers on cell adhesion and biocompatibility, chitosan is valued for a different, complementary set of properties rooted in its distinct polysaccharide chemistry. Chitosan is derived by partial deacetylation of chitin, which is a primary component of the exoskeletons of crustaceans such as shrimp and crabs, as well as the cuticles of insects and the cell walls of fungi [36]. The deacetylation process converts chitin to chitosan by removing acetyl groups and forming free amino groups. The molecular structure of chitosan, characterized by glucosamine and N-acetylglucosamine units, is responsible for its versatile chemical and biological properties, making it a promising candidate for various applications, particularly in the biomedical field [37]. Its ability to form hydrogels and films also makes it ideal for creating scaffolds in tissue engineering, where controlled release of bioactive agents and the promotion of cell adhesion are essential for tissue regeneration [38].

Chitosan can effectively mimic the extracellular matrix (ECM), providing a supportive environment for cellular activities, facilitating better integration with host tissues, and promoting healing. Research has demonstrated that incorporation of chitosan into hydrogels can improve mechanical strength, moisture retention, and biocompatibility, underscoring its significance in the advancement of therapeutic strategies for tissue repair and regeneration [39]. Chitosan can naturally degrade into non-toxic byproducts that the body can safely resorb. In addition, its non-toxic nature, which stems from its natural origin, can minimize the risk of adverse reactions and improve compatibility with biological systems [40].

Chitosan’s cationic nature allows it to bind to negatively charged surfaces of microbial cell membranes, leading to increased permeability and eventual cell death [41,42]. This antimicrobial action is effective against a wide spectrum of bacteria, making chitosan particularly useful in wound care products where infection prevention is crucial [43]. Chitosan is also known for its rapid hemostatic properties, which enhance the natural clotting mechanism of the body. This effect is primarily due to the ability of chitosan to interact with blood cells and platelets, facilitating the accelerated formation of blood clots and thereby reducing blood loss. The positively charged chitosan molecules bind with negatively charged components on the surfaces of platelets and red blood cells, promoting aggregation and triggering the clotting cascade. As a result, chitosan has emerged as a promising material for applications in wound dressings and surgical procedures, where adequate hemostasis is critical [44].

Despite its favorable attributes for wound healing applications, chitosan has been limited by inherent shortcomings, namely poor mechanical properties, which have posed serious hurdles to widespread clinical use. In addition, poor mechanical properties, possible toxicity from certain cross-linkers, and the potential for adverse reactions require further research and modification strategies to maximize their performance and safety profile in biomedicine [45].

Recent work has continued to refine both the sourcing and the functional tuning of chitosan for wound-care applications. Because crustacean-derived chitosan can raise allergenicity and supply-chain sustainability concerns, fungal biomass has been investigated as a complementary, lower-allergen source of chitin and chitosan for advanced wound-management materials, including nanoparticles, membranes, hydrogels, sponges, and nanofibrous scaffolds [46]. At the molecular level, both the degree of deacetylation and the molecular weight strongly govern chitosan’s hydrophilicity, degradation rate, and mechanical performance, making these two parameters a key lever for optimizing chitosan-based scaffolds [47]. The same molecular parameters also influence chitosan’s procoagulant efficiency: a moderate degree of deacetylation combined with a high molecular weight has been reported to yield the most pronounced hemostatic effect, providing a theoretical basis for designing more effective chitosan-based hemostatic dressings [48]. More broadly, the diversity of crustacean- and fungal-derived source material itself contributes to batch-to-batch variation in degree of deacetylation and molecular weight, which in turn propagates into inconsistent porosity, degradation rate, and mechanical strength across nominally similar scaffolds; this source-driven variability, rather than the underlying chemistry alone, is increasingly recognized as a central obstacle to standardizing chitosan-based scaffold performance and to their regulatory acceptance [49].

### 3.3. Cellulose: An Abundant Biorenewable Polymer

Rounding out the three materials central to this review, cellulose contributes a structural role distinct from chitosan’s antimicrobial activity or gelatin’s cell-adhesive properties. As the most common biopolymer on Earth, cellulose is a crucial structural part of plant cell walls and can also be synthesized by certain bacteria and algae [50]. Its availability in nature, biodegradability, and renewability increase its substantial potential as a green material for a wide variety of biomedical applications, particularly in wound therapy. Because cellulose contains a high concentration of hydroxyl groups, it can form strong hydrogen-bonding networks within and between substrates, which can improve hydrogel cohesion and adhesion. In addition, cellulose derivatives can be prepared from natural sources such as algae, fungi, bacteria, and plants [50,51]. Beyond its renewability, cellulose’s isolation, crystallinity, and surface chemistry can be precisely tuned through oxidation and other chemical modification strategies, positioning crystalline, pristine cellulose as a scalable, sustainable alternative to conventional plastics across a range of applications [52]. Notably, the specific crystalline allomorph obtained during nanocellulose isolation depends not only on processing conditions but also on the source biomass itself; softwood- and hibiscus-derived nanocelluloses, for example, have been shown to adopt a Cellulose II allomorph under acid hydrolysis conditions that yield the more common Cellulose I allomorph from other sources [53].

Bacterial cellulose (BC), also called bacterial nanocellulose (BNC), is produced by fermentation of certain bacteria, is also a suitable material for use in tissue regeneration. The typical hierarchical structure of cellulose also makes it suitable for processing in different forms, including microcrystalline cellulose (MCC), cellulose nanofibers (CNF), and cellulose nanocrystals (CNC) with varying properties and applications [51]. BC has been used in wound care studies because it can help maintain moistness in the dressing, absorb excess exudate, provide mechanical protection, reduce microbial infection, control allergic reactions, relieve wound pain, and remain easy to remove and replace. *Komagataeibacter xylinus* (formerly classified as *Gluconacetobacter xylinus*) is often reported to be a strong producer of bacterial cellulose, and the cellulose produced using this strain has been associated with improved mechanical strength and biodegradability [54].

Microcrystalline cellulose (MCC) typically consists of highly aggregated cellulose microfibrils that are insoluble in water and most organic solvents [55]. Due to its particulate nature, MCC is used primarily as a filler or an excipient in pharmaceutical formulations and as a structural element in composite systems, providing mechanical integrity without the possibility of continuous network formation [56]. Alternatively, cellulose nanofibers (CNF), produced through mechanical fibrillation of cellulose pulp, possess high aspect ratios and can entangle in a network. CNFs have extremely high mechanical strength, large surface area and satisfactory water retention capacity and are therefore suitable for hydrogel formation and as reinforcement fillers in soft matrices [57]. Strong hydrogen bonding networks between CNFs are responsible for their resilient mechanical behavior, can replicate aspects of the ECM, and can create a desirable microenvironment for cell proliferation and adhesion [58].

CNC derived from acid hydrolysis of cellulose to remove amorphous regions, are highly crystalline rod-shaped nanoparticles of typical nanometer-sized dimensions [59]. The specific acid used and the strength of the hydrolysis conditions strongly influence the resulting crystallinity, thermal stability, and fiber morphology of the isolated cellulose, as demonstrated for cellulose extracted from wheat straw via hydrochloric, nitric, and sulfuric acid hydrolysis [60]. Subsequent chemical functionalization of this acid-hydrolyzed wheat-straw cellulose has similarly been characterized, providing further insight into how these processing choices shape the material’s final properties [61]. CNCs are characterized by high crystallinity, stiffness, and high surface area, making them excellent reinforcement elements in composite materials [62]. Their ability to form stable colloidal suspensions and their innate biocompatibility make CNCs useful for enhancing the mechanical strength of hydrogels and scaffolds and to address the universal problem of limited mechanical strength in many natural polymer-based wound dressings [63,64].

The biomedical functions of cellulose and its nanostructured forms are varied. Their inherent hydrophilicity gives them a high water-holding capacity, which is crucial to maintaining a wet wound environment that is important for improved healing and reduced scar formation [65]. Moreover, the high stiffness and mechanical strength of CNF and CNC confer reinforcement in composites and provide the ability to create tunable wound dressings that can withstand exogenous forces but still promote tissue regeneration [66]. Cellulose-based materials such as films, hydrogels, and scaffolds are frequently used in wound care and integrated into composite systems to take advantage of their reinforcing properties [67]. This reinforcing effect is well established beyond biomedical applications as well; for example, incorporation of microcrystalline cellulose into acrylonitrile butadiene styrene (ABS) plastic composites has been shown to significantly improve thermal stability relative to the neat plastic, illustrating cellulose’s broader potential as a sustainable reinforcing filler [68].

However, nanoparticle aggregation and the challenges of achieving uniform dispersion within polymer matrices must be handled with care during the fabrication process [69]. Surface modification strategies such as silanization can improve nanocellulose dispersion within composite matrices, though such modifications must be balanced against their potential impact on the material’s biodegradability [70]. In addition, it is still difficult for existing hydrogel dressings to achieve a desirable balance between mechanical strength, moisture retention function, and biocompatibility.

Beyond the forms discussed above, cellulose chemistry has been further diversified to broaden its biomedical utility. The seminal review by Klemm et al. established the foundational classification of nanocelluloses, including microfibrillated cellulose, nanocrystalline cellulose, and bacterial nanocellulose, which underpins much of the subsequent literature on nanocelluloses in tissue engineering and wound care [71]. Within the cellulose nanofiber family, TEMPO-mediated oxidation has become a particularly important production route, introducing C6 carboxylate groups on the fibril surface that promote spontaneous nanofibrillation and produce individualized high-aspect-ratio nanofibers with high tensile strength [72]. Clinically oriented reviews have further cataloged the growing range of cellulose-based wound-dressing formats, including films, hydrogels, foams, and nanofiber mats, and their comparative performance in protecting the wound bed, absorbing exudate and modulating inflammation [73]. Cellulose-based scaffolds have also drawn attention for their hemostatic potential, since their surface chemistry, porosity, and mechanical properties can be engineered to promote platelet activation and clot formation, positioning cellulose alongside chitosan as a candidate hemostatic biomaterial for wound-care applications [74] (see Table 1).

## 4. Synergistic Potential of Chitosan, Gelatin, and Cellulose

The synergistic combination of chitosan, gelatin, and cellulose has attracted significant attention because these biopolymers can complement each other. Each polymer offers unique capabilities: chitosan for porosity, antimicrobial activity, and hemostatic properties [75], gelatin for improved cell adhesion [21], and cellulose for mechanical reinforcement [54]. When combined, these materials form composites with enhanced performance that helps overcome the limitations of the individual components. However, the greatest challenge remains the precise control of critical material parameters, including mechanical strength, ideal porosity, and a controlled degradation rate, which are necessary for effective wound-healing outcomes.

**Figure 3:**
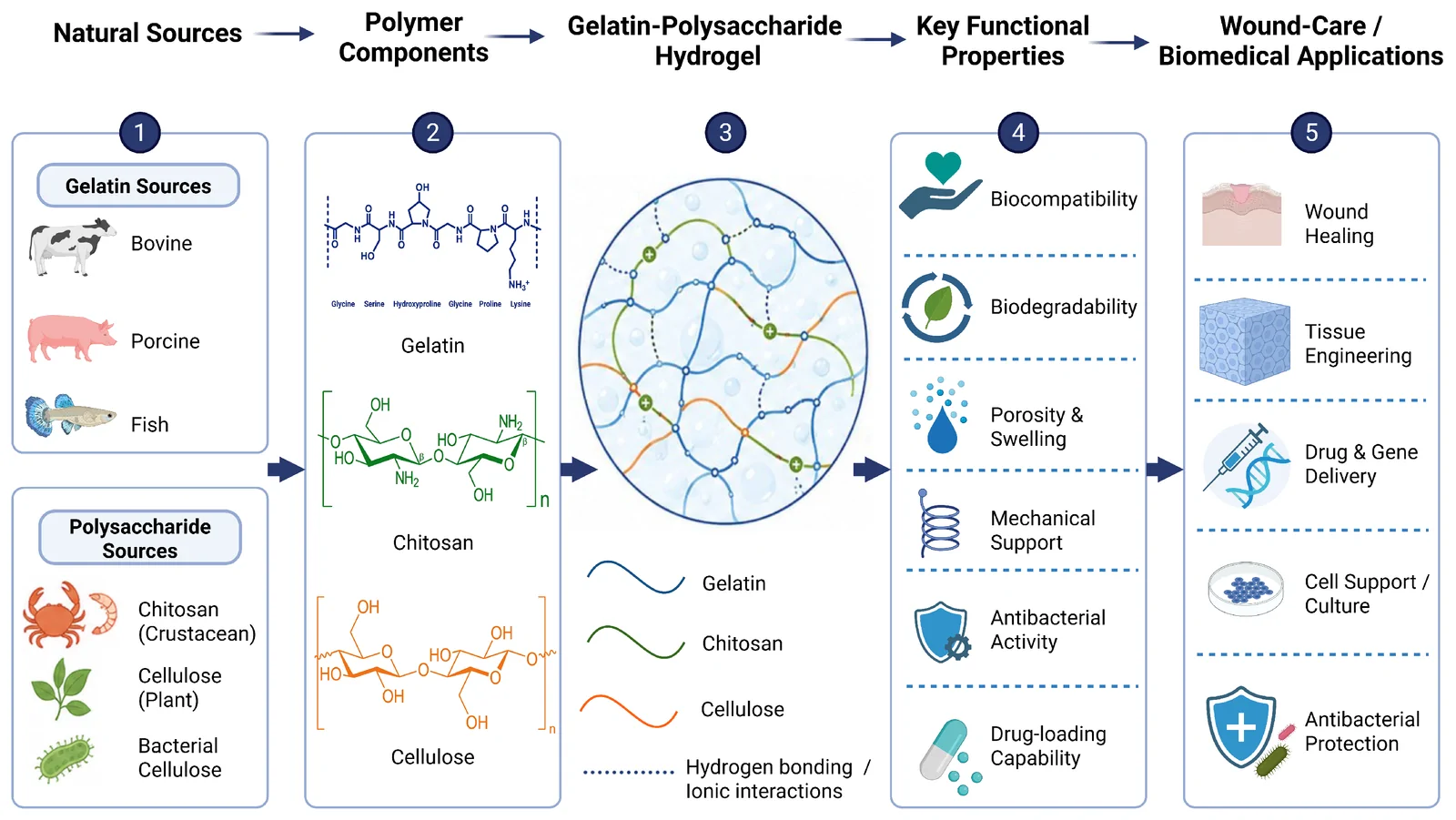
An overview of the origin and significance of hydrogels prepared from gelatin and polysaccharides, along with their biomedical applications. Created in BioRender. King, N. (2026) https://BioRender.com/aj1kyl3.

Achieving this balance often relies on blending gelatin with polysaccharides such as chitosan and cellulose, which are themselves sourced from diverse animal, plant, and microbial origins, including crustacean shells, forest biomass, and bacterial fermentation [76]. Recent studies have expanded gelatin–polysaccharide systems from wound healing [77] and cell growth [78] to applications that inhibit bacterial growth and enable drug, gene, siRNA, and peptide delivery (Figure 3) [79]. These hydrogels can absorb large amounts of water, typically more than 100 times their dry weight, supporting in vitro culture systems for studying mammalian-cell behavior in a matrix-like tissue-engineering environment that encourages cell attachment, growth, infiltration, and vascularization (Figure 2) [80]. At the molecular level, the carbohydrate–protein interactions that support this hydrogel formation can be designed through two major covalent-bonding reactions that emulate ECM proteoglycan [80]: (i) Schiff base formation [81] and (ii) Maillard reaction [76].

Gelatin and chitosan hydrogels, while highly biocompatible and cell-supportive, are typically not mechanically robust and often show poor structural integrity. As a result, they may not be able to support dynamic or load-bearing wound regions [21,82,83]. In these systems, nanostructured cellulose, including cellulose nanofibers (CNF), CNC, and bacterial cellulose (BC), can act as an effective reinforcement material because of its high crystallinity, high aspect ratio, hydrogen-bonding capacity, and ability to form mechanically supportive networks [59,62,71,72]. The crystallinity and high aspect ratio of CNC and CNF can create a robust percolated network within the composite, leading to large increases in compressive modulus and tensile strength without compromising biocompatibility [83]. For example, Li et al. demonstrated that a dual-network chitosan/cellulose nanofiber composite prepared by in situ enzymatic catalysis showed a remarkable improvement in both tensile strength and elongation at break, providing a route to improve strength and toughness at the same time [84]. Similarly, Wegrzynowska-Drzymalska et al. explored dialdehyde cellulose nanocrystals (DCNC) as a crosslinker to chemically reinforce chitosan–gelatin films, improving mechanical properties and adding anti-inflammatory advantages; however, balancing crosslinking density with potential cytotoxicity remains challenging [85]. Furthermore, Bhatnagar et al. reported that CNCs can enable sustained release of platelet lysate in a chitosan–cellulose nanocrystal hydrogel, which in turn enhanced fibroblast proliferation and wound closure in vitro [86].

Studies by Afewerki et al. and Ooi et al. also found that the addition of oil palm-derived nanocellulose improved mechanical characteristics and crystallinity in collagen-nanocellulose scaffolds [87,88]. For a gelatin-based wound dressing to be effective, degradation must be precisely tuned to match the natural pace of tissue regeneration. The rapid degradation of gelatin, which is a drawback in one-component dressings, can be controlled in a composite system; by blending it with slower-degrading chitosan and cellulose, the degradation rate can be adjusted to better match the rate of new tissue growth so that the dressing remains an effective scaffold during healing [89]. In addition, Muzzarelli (2009) utilized the naturally occurring crosslinker genipin to stabilize chitosan/gelatin blends, where thermal degradation, surface wettability, dissolution, and swelling depend on both the composition of the blend and the concentration of genipin used for crosslinking [90].

Beyond mechanical reinforcement, chitosan is also used to tune the porosity of the scaffold in gelatin/cellulose composites. Porosity is another key requirement for tissue-engineering scaffolds, particularly when chitosan is used for structural support and to promote cell proliferation. The scaffold should provide sufficient surface area for cells to thrive with an ideal pore size that allows cell growth and cellular penetration. The objective is a highly interpenetrating pore structure that allows nutrient transport to support growth [91]. The optimum pore size for wound-healing scaffolds varies considerably, but generally falls in the 20 to 300 μm range, depending on tissue type, biomaterial, and application. Smaller pores promote cell attachment, while larger pores (60–300 μm) facilitate cell migration, vascularization, and nutrient transport; for skin regeneration, 20–125 μm is commonly cited as optimal [92].

In gelatin–chitosan–cellulose composites, chitosan can be used to manage porosity by forming a complex with gelatin and cellulose. The optimal porosity reported typically ranges from 60% to 90%, with pore sizes of 50 to 250 μm. Charge interactions between chitosan and the other polymers can be regulated to create interconnected porous networks suitable for nutrient transport, gas exchange, and cell penetration [40]. In a study of the gelatin–chitosan scaffold by Han et al., the porosity of the compound increased with the addition of viscous chitosan [3]. The lowest porosity (only gelatin) was shown by 52.81 ± 13.99%), C10CNC (only chitosan) showed the highest porosity (78.42 ± 9.49%), and intermediate ratios such as G3C7CNC also exhibited higher porosity (67.37 ± 9.09%) [89]. Together, the synergistic combination of these polymers in a composite scaffold can offer additional benefits while reducing their respective shortcomings [89]. Table 2 summarizes the main benefits reported for these composite combinations.

## 5. Fabrication Techniques for Wound Dressing

Realizing the composite benefits described above in a usable wound dressing depends on how these materials are physically processed into a scaffold. The fabrication method chosen strongly influences the resulting pore structure, mechanical behavior, and degradation profile, and several established techniques, discussed below, have been applied to chitosan-, gelatin-, and cellulose-based systems.

### 5.1. Hydrogels

#### 5.1.1. Introduction

A hydrogel is a water-swollen polymer network that can be formed from natural or synthetic polymers. Polymers that compose a hydrogel are cross-linked using different methods, and the selected cross-linking strategy largely determines the final function and performance of the material. In general, cross-linking can be categorized as physical or chemical. Physical cross-linking relied mainly on intermolecular interactions such as hydrogen bonding, ionic interactions, crystallization cross-linking, and hydrophobic association. Physically cross-linked hydrogels are often valued for their high biocompatibility and low molecular toxicity. Chemical crosslinking typically occurs through covalent bonding between polymer chains (e.g., free-radical polymerization or radiation crosslinking). As a result, chemically crosslinked hydrogels generally show improved mechanical properties, and chemical crosslinking is a common approach to enhance hydrogel mechanical strength [91]. Hydrogels have played a central role in tissue engineering because they can provide structural mimics of ECM biomacromolecules, support cell functions, and allow the permeability of oxygen, nutrients, and other water-soluble metabolites [3,40,92]. In aqueous environments, hydrogels absorb and retain water (swell), making them a useful class of biomaterials for 3D cell culture.

#### 5.1.2. Hydrogels for Wound Care

In particular, hydrogels have been a preferred option for wound dressings and for promoting chronic wound healing. In wound healing and soft tissue engineering, hydrogels can easily conform to irregular wound shapes while maintaining moisture and absorptive function. They are also flexible and can be further designed through chemical modifications to reinforce their properties or introduce new biological functions.

For example, Kanimozhi et al. prepared a chitosan/poly(vinyl alcohol)/methylcellulose (CS/PVA/MC) hydrogel scaffold with substantial potential for wound healing. The hydrogel showed biocompatibility and non-toxicity and supported the proliferative functions of fibroblast cells that are critical to tissue healing. Due to its chitosan content, the material also exhibited inherent antibacterial action against common wound pathogens such as *S. aureus* and *E. coli*. In its hydrated form, the scaffold functioned as a hydrogel with structural properties suitable for use as a wound dressing. Its highly porous, connected morphology and high swelling capacity allowed it to manage wound exudate while also providing routes for transport. The inclusion of methylcellulose further enhanced hydrogel flexibility. In general, the combination of biocompatibility, antibacterial efficacy, high absorbency, and flexibility makes this CS/PVA/MC hydrogel scaffold a strong candidate for next-generation wound dressings [93].

A nanocomposite hydrogel was also developed by Tanpichai and Oksman through reinforcement of a PVA matrix with CNCs. The purpose of this hydrogel was to improve performance in biomedical and tissue engineering applications, and it showed good elasticity and recovery after deformation, which are crucial for applications such as artificial cartilage or wound dressings. With greatly improved mechanical strength and elastic recovery while still maintaining high water content, approximately 92%, the cross-linked CNC–PVA hydrogel was reported to be a promising material for demanding biomedical applications [94].

In a review by Adelnia et al., PVA hydrogels prepared by the physical freeze–thaw (F–T) process were described as versatile biomaterials due to their unique fabrication pathway and tunable properties. A major advantage of the F–T process is that it can form highly biocompatible and ultrapure hydrogels without the need for chemical cross-linking agents, which makes these systems well suited for biomedical applications. Pure PVA hydrogels provide a wet protective environment, but are often combined with active compounds such as chitosan, alginate, or silver nanoparticles to introduce antimicrobial and wound-stimulatory properties [95].

Figueroa-Pizano et al. also used the F–T method to prepare chitosan–poly(vinyl alcohol) (CS–PVA) hydrogels without relying on potentially toxic chemical cross-linkers. Their study examined how adjusting F–T conditions, including freezing temperature and number of cycles, can modulate the physical structure and drug-release profile of the hydrogel. A key observation was that porosity could be controlled by changing the freezing temperature; SEM micrographs and porosimetry showed that reducing the freezing temperature, from −4 °C to −80 °C, produced smaller pore sizes but greater total porosity. This occurs because lower temperatures promote the rapid formation of many small ice crystals, which, upon sublimation, leave behind a more porous network [96].

Moreover, Koosha et al. combined honey and allantoin with physically crosslinked hydrogel films composed of chitosan and PVA for wound healing applications. The resulting hydrogel showed beneficial properties for wound healing, including long-term biocompatibility, antimicrobial activity, and appropriate and accelerated wound closure in in vivo models [97].

Mansur et al. engineered a bioadhesive hydrogel as a therapeutic dressing for chronic wounds by optimizing bioadhesion to improve cellular interaction. They reported the design, synthesis, and characterization of hydrogels obtained from carboxymethylcellulose (CMC) and PVA, which were chemically crosslinked using citric acid (CA) through a biocompatible thermal treatment process. This approach created a stable hybrid network, and the resulting hydrogel displayed a combination of physicochemical and biological properties that support its potential for next-generation wound care [98].

A ternary hydrogel was synthesized by Fan et al. for wound dressing applications by mixing chitosan, gelatin (Gel), and PVA. The goal was to improve the hemostatic and mechanical properties of gelatin/PVA hydrogels by incorporating chitosan as a hemostatic component. The experiment demonstrated that the resulting CS/Gel/PVA hydrogel achieved a satisfactory balance of wound-dressing attributes, particularly blood clotting performance and mechanical strength. The addition of chitosan significantly increased mechanical strength compared to a regular Gel/PVA hydrogel; however, elongation and tensile strength decreased as the proportion of chitosan increased, because the hydrogel became hard and brittle at high concentrations of chitosan [99].

Another study by Shamloo et al. used the F–T method and incorporated various concentrations of honey into hydrogels formed from PVA, chitosan, and gelatin. They reported that honey addition can provide strong therapeutic effects in wound healing. In vivo experiments in rat models showed enhanced wound closure, with contraction approaching 95% on day 12. Honey also improved antibacterial efficacy against *S. aureus* and *P. aeruginosa* and increased fibroblast viability, although these benefits were coupled with trade-offs in physicochemical properties. Acting as a plasticizer, honey increased flexibility and maximum strain while reducing ultimate tensile strength and elastic modulus; its high solubility also increased scaffold degradation [100].

Together, these hydrogel examples show that wound-dressing hydrogels can be engineered to support moisture retention, antimicrobial activity, controlled bioactivity, and improved mechanical performance, depending on the selected polymer system and fabrication strategy.

### 5.2. Electrospinning

**Figure 4:**
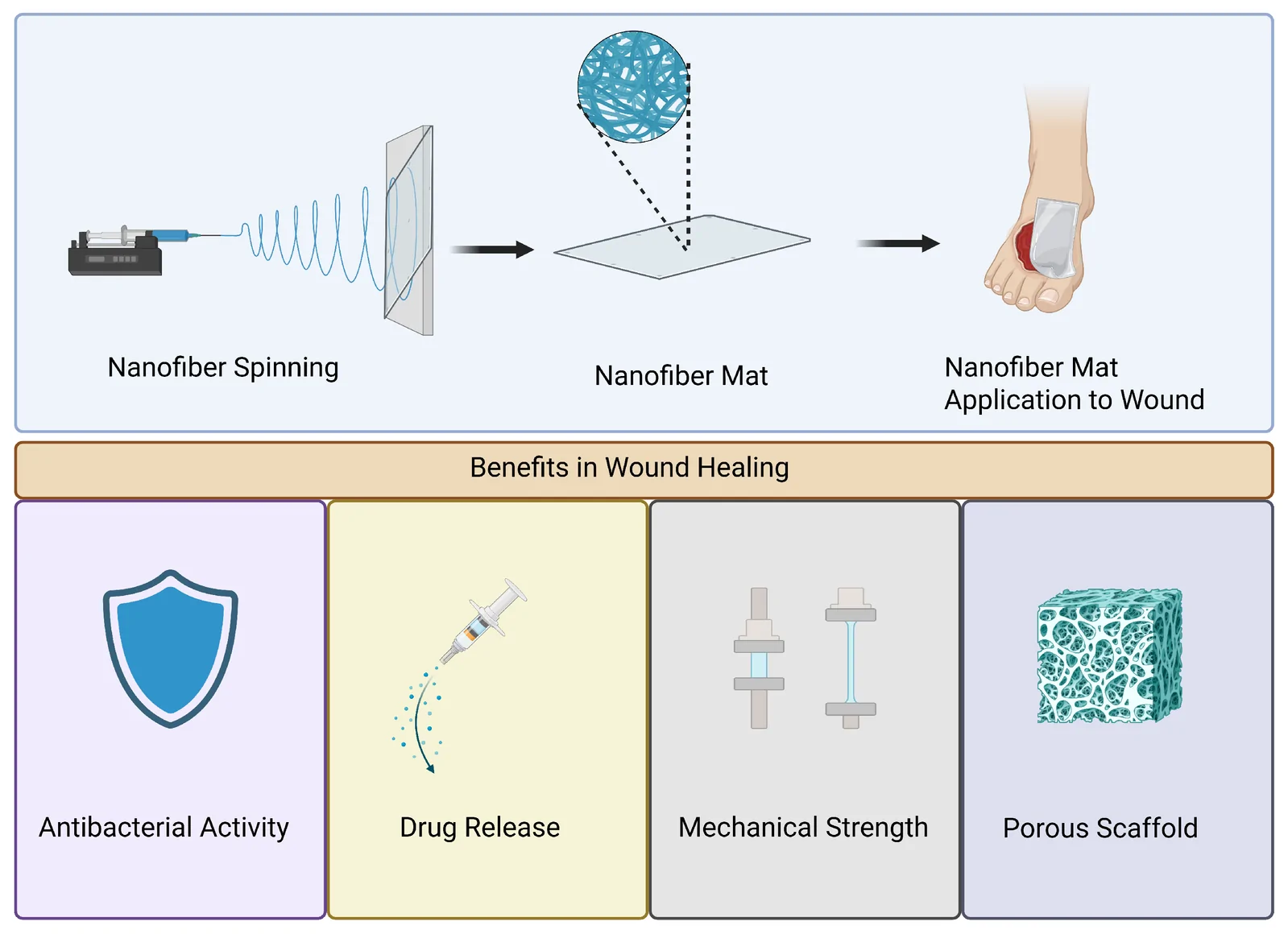
Schematic diagram showing electrospun nanofibers for diabetic wound healing. Created in BioRender. King, N. (2026) https://BioRender.com/stuoi41.

Where hydrogels rely on a swollen, crosslinked network to achieve these properties, electrospinning takes a different structural approach, using an electrohydrodynamic process to create fine, nanofiber-based wound dressings from polymer solutions or melts. Figure 4 illustrates the electrospinning process and how it can be applied in the design of new wound dressings for chronic diabetic wounds. In this process, a polymer solution is pumped through a spinneret and drawn into ultra-thin nanofibers under a high electric field, and the nanofibers are then collected to form a dressing. Once applied to a diabetic wound surface, electrospun dressings can provide antibacterial activity to combat infection, promote faster healing, enable drug delivery over extended periods, and enhance mechanical performance. In addition, the resulting porous scaffold can support cell attachment, proliferation, and tissue regeneration by mimicking key features of the ECM of the body [101].

Single-layer dressings conventionally used in practice may not meet all clinical requirements, and the growing demand for functional wound dressings has attracted interest in bilayer designs [102]. Differences in structure and properties between layers can confer several advantages. For example, a denser outer layer can protect the wound against infection and mechanical stress while minimizing dehydration, whereas the inner layer (in direct contact with the wound) can be designed to mimic the ECM and promote cell adhesion and proliferation [103]. In general, combining functionalities within bilayer dressings shows the potential to advance wound care management and improve patient outcomes [104].

A study by Ghafari et al. investigated a bilayer wound dressing consisting of a dense polyurethane/ethanolic extract of propolis (PU/EEP) membrane as the top layer and a polycaprolactone/gelatin (PCL/Gel) scaffold electrospun as the sublayer. The upper layer was designed to impart antimicrobial activity and protect the wound from external contaminants, while the sublayer provided a cell-friendly scaffold. Both layers showed good biocompatibility (non-toxic and cell-viable), and the PCL/Gel scaffold can biodegrade over, potentially avoiding removal after healing. In this bilayer configuration, faster wound healing can be supported through increased cell adhesion and proliferation; however, rapid degradation of gelatin can affect structural stability, which was addressed by combining gelatin with PCL [105].

In a recent study, H. Samadian et al. applied graphene nanosheet-reinforced, electrospun chitosan/gelatin nanofiber scaffolds for wound healing and infection prevention. They reported high porosity (~90%), which supports fluid absorption and biodegradability, as well as strong antibacterial effects (reported reductions of ~50% and ~80% for *E. coli* and *S. aureus*, respectively). The scaffolds did not exhibit toxicity to human cells and improved cell motility to support faster wound closure; however, at high graphene content, bead formation problems and reduced porosity were observed [106].

Kazeminava et al. also reported electrospun wound dressings based on gelatin and chitosan nanofibers, loaded with a phlorotannin-rich extract of *Undaria pinnatifida*, aiming to emulate antibacterial and anti-inflammatory functions. The fibers were highly porous (~90%), were permeable to water vapor, and allowed controlled release of the extract for up to 160 days, which supports long-term use in wound care. Antibacterial activity was effective against *Pseudomonas aeruginosa*, while *Staphylococcus aureus* showed higher resistance; the main limitations were reduced effectiveness against *S. aureus* and relatively weak mechanical properties [107].

Furthermore, Haghbin et al. assessed electrospun polyurethane–gelatin nanofibrous membranes loaded with honey and zinc oxide nanoparticles for wound dressing applications, focusing on antibacterial properties, mechanical strength, and biocompatibility. PU/Gel/H/ZnO membranes showed strong antibacterial effects, with clear inhibition zones against *E. coli*, *S. aureus*, and *Bacillus subtilis*. However, while gelatin contributes to good biocompatibility and a hydrophilic surface, its fast degradation remains a limitation for long-term applications [5].

The synergistic combination of gelatin (GEL) and cellulose acetate (CA) in electrospun nanofibers is also considered an interesting biomaterial approach for future wound dressings. Cellulose acetate can provide the required mechanical support and structural integrity to help the scaffold maintain its structure, while gelatin contributes biocompatibility and bioactivity that replicate the native extracellular matrix and support cell adhesion, proliferation, and migration. These CA/GEL nanofibers can form highly porous scaffolds with a well-interconnected architecture and high surface-area-to-volume ratio, which can improve gas exchange, regulate wound exudate, and help establish an optimal microenvironment for cellular infiltration and tissue regeneration, thus accelerating the healing cascade [106].

Finally, electrospun nanofibers of CMC and PVA have also been used to fabricate antimicrobial wound dressings with improved mechanical properties and loading of antibiotics such as colistin (CL). These systems were reported to show suitable biocompatibility with (more than 80% viability in human skin cells) and improved antibacterial activity against pathogens such as *S. aureus* and *E. coli*. In addition, the nanofibrous scaffold can be designed to replicate the ECM of the skin and facilitate the migration and proliferation of cells needed for healing [107].

### 5.3. Freeze-Drying

**Figure 5:**
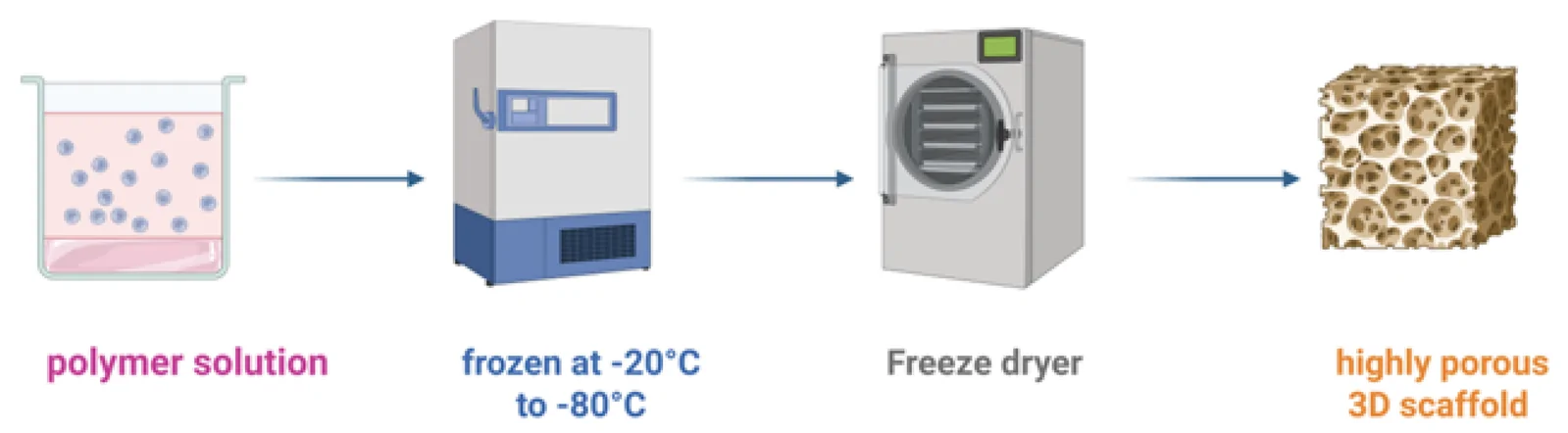
Freeze-drying process. Created in BioRender. King, N. (2026) https://BioRender.com/j8j4nc0.

Freeze-drying offers a further route to a highly porous scaffold, converting a polymer solution into a 3D structure with porosity levels of more than 90% commonly reported. In general, the freeze-drying technique includes three steps: (i) preparation of the polymer solution, (ii) freezing (typically from −20 to −80 °C) and (iii) lyophilization of the frozen system reduced under pressure to generate an interconnected porous scaffold [5,108] (Figure 5).

In one study, the authors focused on chitosan–gelatin scaffolds loaded with copper nanoparticles for skin tissue engineering. Poor mechanical strength and rapid degradation limit the use of gelatin alone in these systems. The value of chitosan lies in its biodegradability, antimicrobial activity, and hydrogel formation capability, which makes it useful for skin regeneration; however, compared to gelatin, chitosan can be brittle and shows limited cell adhesion. The combination of chitosan and gelatin can therefore provide enhanced biocompatibility and structural features that promote cell growth. The addition of copper nanoparticles further increased porosity and mechanical stability, although at high loadings the scaffold tended to compact and porosity decreased [109]. Overall, the chitosan–gelatin blend still needs to be fine-tuned, particularly due to the rapid degradation of gelatin and the stiffness of chitosan.

Han et al. made Gel/Chi sponges by freeze-drying and reported a uniform porous structure with pore sizes in the range of 120–140 μm and porosity greater than 90%. This indicates that the scaffold can be suitable for cell seeding and nutrient transport. Water uptake was higher (more than 1500%) and water retention was also notable; however, the degradation rate increased linearly with the higher gelatin content, which may raise concerns about long-term stability. Although overall biocompatibility was good, cell proliferation decreased with higher chitosan content, which was attributed to excessive cellular electrostatic interactions [3].

In another study, chitosan–gelatin scaffolds were developed for dermal tissue engineering using freeze-drying. A 2:5 chitosan-to-gelatin weight ratio provided a balance of mechanical and cellular properties; scaffolds supported cell proliferation, but still faced challenges such as brittleness and rapid degradation [4].

Wang et al. investigated a wound dressing formulation based on gelatin and chitosan, each reinforced with a piece of nonwoven fabric, and incorporated *Centella asiatica* extract into the composite hydrogel. The hydrogel was designed as a biocompatible antibacterial dressing that may help counteract the growth of antibiotic resistance. However, incorporation of chitosan tends to reduce elasticity and increase pore size, which can affect structural integrity; its hydrophobic nature can also negatively affect swelling capacity [110].

Zulfiqar et al. reported chitosan-based wound dressings loaded with copper metal–organic frameworks (Cu-MOFs) to enhance antibacterial and wound healing properties, support angiogenesis and collagen deposition, and accelerate wound healing in a rat model infected with *Pseudomonas aeruginosa* [111].

Ghosh et al. prepared and characterized bio-scaffolds based on carboxymethyl chitosan, gelatin, and copper oxide nanoparticles. These scaffolds were fabricated by freeze-drying followed by two crosslinking steps: an initial crosslinking using CuO nanoparticles and a secondary crosslinking using epichlorohydrin in an ammonia-rich atmosphere to enhance water resistance and mechanical stability. The authors evaluated swelling, hydrolytic degradation, moisture transmission, surface roughness, and antibacterial performance, and reported that crosslinking improved structural stability and mechanical properties while maintaining antibacterial activity against Gram-positive and Gram-negative bacteria [112].

### 5.4. Salt Leaching

**Figure 6:**
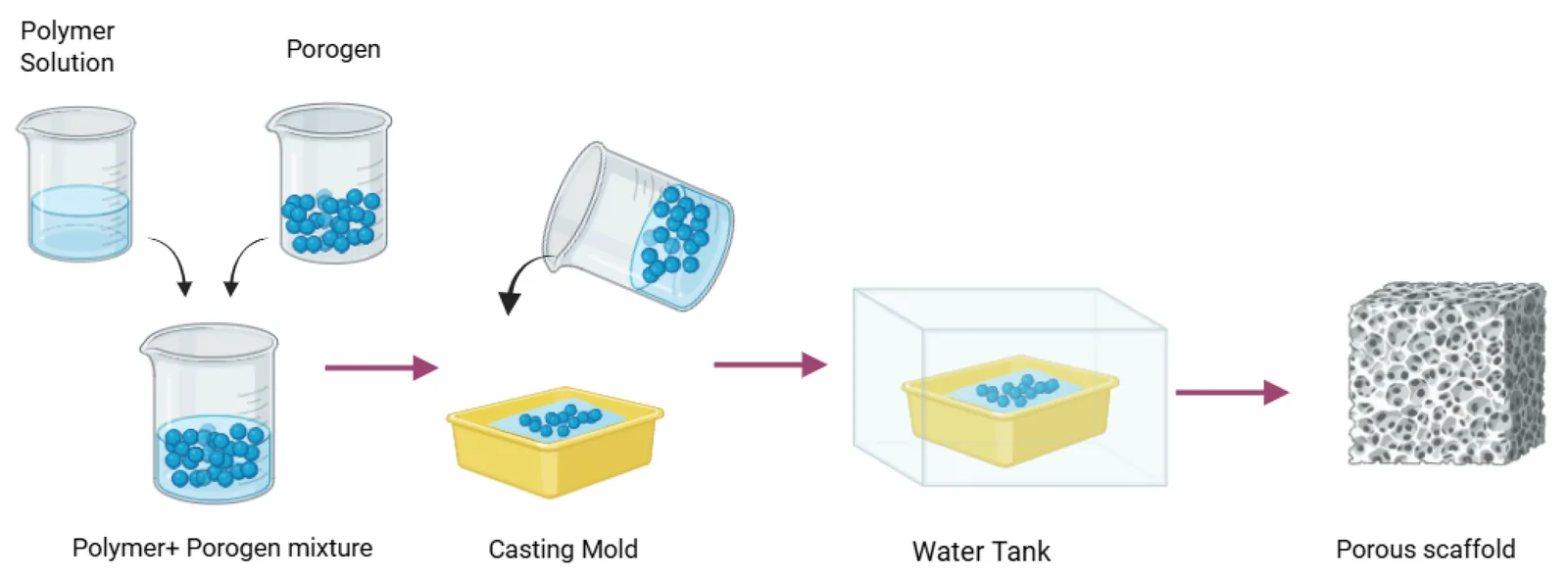
A schematic workflow of the preparation of porous scaffolds via porogens. Created in BioRender. King, N. (2026) https://BioRender.com/wydnk5g.

In contrast to the more equipment-intensive freeze-drying and electrospinning processes, salt leaching is a simple and widely used method for forming 3D porous scaffolds, and it can provide an appropriate microenvironment for cell growth due to the ease of creating interconnected pores. This technique can offer enhanced mechanical support and physical and biochemical stimuli that support cell proliferation. In salt leaching, pores are produced using porogens (soluble particles such as sugar, salt, wax, paraffin, or gelatin) [113,114]. Figure 6 shows a schematic workflow for preparing porous scaffolds through porogens: Porogens are mixed into a polymer solution, the mixture is cast in a mold, and the porogen is then dissolved (typically in water), leaving behind a porous structure [115].

The salt leaching technique was introduced by Mikos et al. [116] and mainly applied to prepare composite scaffolds; later, it also found applications in porous scaffolds designed for the growth of endothelial cells.

A commonly described procedure involves the preparation of a 25% gelatin solution mixed with NaCl crystals, with an average particle size of 300–500 μm. The mixture is poured into a mold and dried (e.g., in a vacuum oven at 50 °C), followed by cross-linking in an acetone/water mixture containing 1-ethyl-3-(3-dimethylaminopropyl)carbodiimide (EDC). After cross-linking, excess NaCl is washed out with water, leaving uniformly distributed, interconnected macropores with sizes that reflect the original NaCl particle size. One key issue is that insufficient NaCl can lead to a bilayer structure, where a dense layer forms at the bottom due to the sinking of the salt particles. In addition, although salt leaching is effective in creating porosity, an excessive NaCl content does not necessarily improve mechanical properties and may compromise the integrity of the scaffold [117].

Morpara and Wiwatwongwana investigated gelatin/CMC scaffolds fabricated by salt leaching, where gelatin and CMC were dissolved in distilled water and NaCl was used as the porogen. The Scanning Electron Microscopy (SEM) analysis showed interconnected porous structures with pore sizes ranging from 113.46 to 212.82μm, and the pore size decreased with increasing CMC content. Although salt leaching is straightforward and inexpensive, it still lacks definite control over the pore-size distribution [118]. Kanimozhi et al. also employed salt leaching to fabricate chitosan/poly(vinyl alcohol)/methylcellulose –ZnO (CS/PVA/ MC3 –ZnO) nanocomposite scaffolds designed for tissue engineering applications. In this approach, sodium chloride particles served as a porogen, resulting in an interconnected porous architecture. The resulting high-porosity structure was advantageous for cellular infiltration and growth, and the reported physicochemical and biological properties were conducive to wound healing applications in skin tissue engineering [93].

### 5.5. 3D Printing

Whereas hydrogel casting, electrospinning, freeze-drying, and salt leaching each offer some degree of control over scaffold architecture, 3D printing, also known as additive manufacturing, pushes this control further and is considered a new frontier in scaffold fabrication. This technology builds patient-specific three-dimensional structures layer-by-layer directly from a digital model. Unlike traditional approaches, such as solvent casting or freeze-drying, where definitive control over pore geometry may not be readily achievable, 3D printing enables hydrogels with complex, pre-designed microporous structures to be fabricated in a timely and accurate manner, supporting better mimicry of the natural ECM. This level of control is highly crucial for improved cell proliferation, improved material transport, and, subsequently, tissue regeneration [119].

For example, Hafezi et al. showed that chitosan can be successfully 3D printed using an extrusion-based method to develop skin tissue-regenerating scaffolds. In that work, 3D-printed chitosan film matrices were crosslinked with genipin to produce flexible dressings. However, in their non-plasticized state, the chitosan films were brittle and showed a low elongation at the break (2.44%). Therefore, plasticizers such as polyethylene glycol (PEG) were added and an optimized film was obtained with much higher flexibility (22.67% elongation at break) [120].

Gelatin is also one of the most common biopolymers harnessed for bio-inks, and one strength of 3D printing is the ability to combine materials to create more functional dressings. For example, one study fabricated an antibacterial 3D-printed patch using an extrusion-based process with a gelatin bio-ink that incorporates Manuka honey (MH). In that study, the addition of MH enhanced the printability, print resolution, and structural fidelity of the gelatin hydrogel. The resulting 3D Manuka–gelatin patches were printable and showed promising antibacterial activity against both *S. aureus* and *E. coli* [121].

Among all these materials, cellulose is an ideal bio-ink component, especially in its nanostructured forms (CNC and CNF), due to its ability to form networks that mimic ECM and provide mechanical reinforcement [122]. In another study, cellulose nanofibrils were combined with casein and chitosan to create a 3D composite scaffold. In that system, TCNFs provided hydrophilicity for blood absorption, while chitosan acted as a cross-linking agent for structural reinforcement [123]. Table 3 provides a comparison of the main fabrication techniques used in the development of wound-healing scaffolds.

## 6. Translational and Manufacturing Considerations

Despite substantial laboratory-scale progress, translating chitosan-, gelatin-, and cellulose-based scaffolds into clinically deployed wound-care products faces several practical barriers that are not always addressed in the primary literature discussed above.

*Manufacturing scalability.* Many of the fabrication techniques discussed in this review, particularly electrospinning and 3D printing, are optimized at the laboratory bench scale. Scale-up to industrial production volumes under Good Manufacturing Practice (GMP) conditions can introduce batch-to-batch variability in fiber diameter, pore architecture, and mechanical properties that is rarely characterized in the studies cited above [123].

*Sterilization.* Standard terminal sterilization methods, such as gamma irradiation, ethylene oxide exposure, and autoclaving, can degrade the mechanical integrity and bioactivity of natural polymers such as gelatin and chitosan; for example, gamma irradiation has been shown to alter the morphology and strain-at-break of chitosan wound-dressing membranes, whereas ethylene oxide exposure preserved these properties more consistently [124]. Few of the studies discussed in this review report how sterilization affects final scaffold performance, representing an important gap for translational development.

*Batch-to-batch reproducibility.* Natural polymers are inherently variable in molecular weight, degree of deacetylation (for chitosan), and source purity (e.g., mammalian- versus fish-derived gelatin, or crustacean- versus fungal-derived chitosan), which can affect crosslinking efficiency and final scaffold properties. This variability is a recognized but underreported challenge across the literature reviewed here, and has been identified as a key barrier to regulatory acceptance and standardization of chitosan-based scaffolds more broadly [123].

*Regulatory considerations.* Wound-care scaffolds incorporating natural polymers are typically regulated as medical devices, for example under the U.S. FDA 510(k) pathway or the EU Medical Device Regulation (MDR). Regulatory approval requires standardized characterization of degradation products, biocompatibility testing (per ISO 10993, Part 1), and demonstrated manufacturing consistency, requirements that extend well beyond the in vitro and small-animal in vivo studies emphasized in most of the literature discussed above [125]. Importantly, the specific regulatory classification and evidentiary burden depend on the final product design, intended use, and any incorporated active components: a plain polymer scaffold, a scaffold combined with an antimicrobial or other bioactive agent, and a substance-based device whose primary mode of action is physicochemical rather than pharmacological can each fall under different classification rules and review pathways [126].

Addressing these translational gaps through standardized characterization protocols, scale-up studies, and sterilization-compatible formulations will be essential for moving chitosan-, gelatin-, and cellulose-based wound-care scaffolds from the laboratory toward clinical practice.

## 7. Conclusions

Combining cellulose and chitosan with gelatin has excellent potential to improve scaffold designs in skin tissue engineering, particularly for wound healing. Cellulose provides biocompatibility, biodegradability, and an excellent capacity to absorb and retain fluids, while chitosan can help fight infection, support blood clotting, contribute to high porosity and biodegradability. Gelatin can promote cell attachment and growth. However, improvement is still needed in how these materials can be designed to form uniform and interconnected pores that are essential for cell growth and nutrient transport. This review highlights the importance of porous structure in scaffolds, where highly porous regions support cell adhesion, proliferation, and differentiation, while less porous regions can provide the mechanical strength required for stability. In general, the influence of preparation parameters on the pore structure and the associated biological consequences highlight the need for careful tuning to achieve optimal scaffold performance.

Future research should focus on improving techniques to produce more uniform pore structures while preserving the functionality of the scaffolds in real wound environments. In addition, studying the degradation behavior of these materials over time will be critical to better match the body’s healing process. Future studies should also control scaffold architecture to balance mechanical stability with biodegradability, supported by extended in vivo studies to verify safety and functionality. Only then can these innovations be translated into clinically viable and effective solutions for skin tissue regeneration.

## Figures and Tables

**Table 1:** Comparison of natural polymers commonly used in wound healing scaffolds.

Property	Gelatin	Chitosan	Cellulose
Source	Collagen-derived protein	Deacetylated chitin	Plant or bacterial cellulose
Biocompatibility	High	High	High
Biodegradability	High	High	High
Mechanical Strength	Low	Moderate	High
Antimicrobial Activity	Limited	Excellent	Limited
Hemostatic Activity	Moderate	Excellent	Limited
Cell Adhesion	Excellent	Good	Moderate
Moisture Retention	High	High	High
Primary Limitation	Rapid degradation	Brittleness	Limited bioactivity

Note: Values represent general trends reported across the literature discussed in this review rather than fixed or universal material properties. The properties of each polymer depend strongly on factors such as molecular weight, degree of deacetylation (chitosan), source, crystallinity, chemical modification, crosslinking strategy, and testing conditions; see Section 3 and Section 4 for further discussion of these dependencies.

**Table 2:** Advantages of composite scaffold systems based on gelatin, chitosan, and cellulose.

Composite System	Primary Benefit
Gelatin–Chitosan	Enhanced cell adhesion and antimicrobial activity
Gelatin–Cellulose	Improved mechanical stability and moisture retention
Chitosan–Cellulose	Enhanced strength, porosity, and wound protection
Gelatin–Chitosan–Cellulose	Balanced biocompatibility, mechanical performance, and regenerative functionality

**Table 3:** Comparison of fabrication techniques used in wound healing scaffold development.

Method	Advantages	Limitations	Typical Applications
Hydrogels	Excellent moisture retention; adaptable chemistry	Limited mechanical strength	Chronic wound care
Electrospinning	ECM-mimicking nanofibers; high surface area	Solvent limitations; scaling challenges	Nanofibrous wound dressings
Freeze-Drying	Highly porous structures	Mechanical weakness	Porous tissue scaffolds
Salt Leaching	Simple and inexpensive	Limited pore-size control	Porous scaffold fabrication
3D Printing	Precise architectural control	Equipment cost; bioink constraints	Patient-specific wound dressings

## Data Availability

No new data were created or analyzed in this study. Data sharing is not applicable to this article.

## Abbreviations

The following abbreviations are used in this manuscript:

BC	Bacterial cellulose
BNC	Bacterial nanocellulose
CA	Cellulose acetate
CMC	Carboxymethylcellulose
CNC	Cellulose nanocrystals
CNF	Cellulose nanofibers
CS	Chitosan
ECM	Extracellular matrix
EDC	1-ethyl-3-(3-dimethylaminopropyl)carbodiimide
FT	Freeze-thaw
Gel	Gelatin
GelMA	Gelatin methacryloyl
MCC	Microcrystalline cellulose
PCL	Polycaprolactone
PEG	Polyethylene glycol
PVA	Poly(vinyl alcohol)
SEM	Scanning Electron Microscopy
